# A new twist in metabolic T cell exhaustion

**DOI:** 10.1097/IN9.0000000000000080

**Published:** 2026-04-29

**Authors:** Konstantinos Aliazis, Vassiliki A. Boussiotis

**Affiliations:** 1 Department of Hematology-Oncology, Beth Israel Deaconess Medical Center, Boston, MA, USA; 2 Cancer Research Institute, Beth Israel Deaconess Medical Center, Harvard Medical School, Boston, MA, USA

**Keywords:** cancer immunotherapy, T cell exhaustion, checkpoints, programmed cell death protein 1, metabolism

## Abstract

Metabolic reprogramming of T lymphocytes has a decisive role in their activation, differentiation, and functional fate commitment. Metabolic reprogramming guides not only the generation of T effector and T memory cells but also the development of dysfunctional exhausted (T_EX_) cells. A recent study by Haku et al published in *Nature Immunology* provides new insights into the role of mitochondria in regulating the metabolic capabilities of T_EX_ cells and their implications for antitumor immunity.

During the last decade, the holy grail of cancer immunotherapy is the understanding of the T cell exhaustion (T_EX_) state ^[1]^. This is of vital importance because T effector (T_EFF_) cells induced by programmed cell death protein 1 (PD-1) immune checkpoint immunotherapy are derived from partially exhausted T cells, termed progenitor T_EX_ cells (T_PEX_) that retain functional potential ^[2]^. Although various groups use different phenotypic markers to characterize T_EX_ cells, the current consensus defines CD8^+^ T_EX_ as PD-1^+^TIM-3^+^ cells. Early studies have documented that PD-1 engagement by PD-L1 leads to inhibition of glycolysis and promotion of fatty acid oxidation (FAO) ^[3]^. However, it is unclear how aberrations in glycolysis and FAO in the tumor microenvironment (TME), in the context of checkpoint signaling, regulate the metabolic fate of T cells toward the development of T_EX_. Although it is well established that glycolysis is required for the generation of T_EFF_^[4]^ and FAO supports T memory (T_MEM_) cell differentiation ^[5]^, studies have shown that excessive glycolysis is associated with T_EX_ state ^[6]^, whereas others have attributed T_EX_ development to aberrant mitochondrial metabolism ^[7]^. It is conceivable that the impaired mitochondrial metabolism might drive tumor-infiltrating T lymphocytes (TIL) to enhance glycolysis to meet their metabolic demands, albeit unsuccessfully, leading to the T_EX_ state ^[8]^. The mechanistic link between impaired mitochondrial function and enhanced glycolysis, and the way by which the combination of these metabolic events leads to the T_EX_ state, has not been understood.

In a recent issue of *Nature Immunology*, Haku et al ^[9]^ have identified progressive states of mitochondrial impairment and provided a metabolic link between such mitochondrial states and glycolysis-driven T_EX_ development. The investigators found that despite high levels of PD-1 expression, tumor-infiltrating T_EX_ cells favor glycolysis and decrease reliance on FAO, due to dysfunctional mitochondria, which accumulate active aldehydes as a byproduct of lipid peroxidation. Importantly, exogenous aldehydes, exemplified by acrolein, lead to mitochondrial damage, impaired FAO, and directly enhanced glycolysis concomitantly with activation of the Akt-mechanistic target of rapamycin (mTOR) pathway. Conversely, aldehyde scavenging reversed T_EX_ state and allowed expansion of T_EFF_ cells and improved antitumor function. Acrolein might directly activate glycolysis downstream of Akt-mTOR, as supported by the investigators, but the enhanced glycolysis might also be a consequence of acrolein-induced mitochondrial damage.

Although the role of mitochondrial dysfunction in T_EX_ development was documented before ^[7]^, this study is significant because identified nuance features of mitochondrial impairment based on mitochondrial potential and mitochondrial mass, classifying T_EX_ into four progressive levels of dysfunction (D1–D4). Exhaustion markers such as TIM-3 and lymphocyte activation gene-3 (LAG-3) gradually increased in D1 to D3 populations, whereas Slamf6, the marker of T_PEX_^[1]^, was decreased. Surprisingly, Slamf6 was upregulated in the D4 terminally T_EX_ population. Similarly, to an in vitro model of T_EX_ generation, time-course experiments in an in vivo tumor model showed that T_EX_ TIL populations sequentially increased from D1 to D4, suggesting a transition from D1 to D4, a result which was further investigated by single-cell RNA sequencing. Metabolic tracer studies of TIL cultured in vitro showed that glycolysis was enhanced from D1 to D3 but not in the proapoptotic D4 group. In parallel, the key enzyme for FAO, hydroxyacyl-CoA dehydrogenase A (HADHA), and the carnitine-acylcarnitine translocase SLC25A20 were gradually decreased from D1 to D4, and these correlated with impaired FAO and loose cristae architecture, consistent with impaired mitochondrial function.

To better understand the role of FAO in the development of T_EFF_ versus T_EX_ cells in the TME, Haku et al generated mice with conditional deletion of HADHA in T cells. HADHA-deficient T cells showed no difference in the phenotypic profile or metabolic activity at baseline, but when stimulated in vitro, preferentially utilized glucose instead of mitochondrial metabolism. Furthermore, in the context of MC38 tumor in vivo, HADHA-deficient mice showed an increased number of PD-1^+^TIM-3^+^ and TOX^+^ cells, lower proliferation capacity compared to controls, and were unable to mitigate tumor growth. HADHA-deficient TIL that were unable to activate FAO displayed elevated expression of the fatty acid transporter CD36, increased fatty acid uptake, and higher activation of the Akt pathway. Because in the TME CD8^+^ TIL are reliant on fatty acid metabolism and FAO for their survival ^[10]^, the authors supported that these results explain why T cells with impaired lipid metabolism had increased glycolysis and impaired antitumor function. It should be noted that caution is required in interpreting these observations because in vitro nutrient utilization might not accurately recapitulate metabolic patterns of physiologically activated T cells in vivo. Stable ^13^C-glucose isotope tracing studies have revealed that although T cells activated in culture preferentially utilize glucose for anaerobic glycolysis and lactate production, pathogen-activated T cells in the intact host display predominant usage of glucose for anabolic metabolism ^[11]^. Similar tracing approaches in cancer models indicated that innate and adaptive immune cells as well as cancer cells have differential capacity to uptake and utilize certain nutrients in the TME with myeloid cells displaying the greatest capacity to take up intratumoral glucose, followed by T cells, and cancer cells which predominantly utilize glutamine, highlighting the complex cell-specific metabolic rewiring in the TME in vivo ^[12]^.

Haku et al utilized mitochondrial mass and potential to characterize T cell exhaustion states more precisely compared to conventional phenotypic markers. The different T_EX_ cells do not rely solely on glycolysis or FAO but instead utilize both metabolic pathways for energy demands, depending on the exhaustion state. As the exhaustion state progresses, glycolysis becomes enhanced, whereas FAO is compromised, further promoting T cell exhaustion. It is conceivable that together with environmental nutrient limitation in the TME due to nutrient consumption by the tumor cells, metabolic insufficiency is deepened, driving T cells into an irreversible exhausted state (D4). Notably, nutrient insufficiency in the TME was previously shown to inhibit mTOR and decrease glycolysis ^[13]^. This is in contrast to the observations of Haku et al., who noted that glycolysis is increased during progressive T cell exhaustion in the TME despite competition for glucose. The authors considered that the morphological mitochondrial changes of intratumoral CD8^+^ T cells leading to loosen cristate are a result of T cell exhaustion. This is intriguing because similar mitochondrial morphological changes have been previously identified in T_EFF_ cells ^[14]^. Further studies are required to compare how the T_EFF_ and T_EX_ activation processes alter mitochondrial dynamics, morphology, and metabolic function.

The study by Haku et al advances our knowledge and shifts the known paradigm in T_EX_ biology by discovering that intratumoral T cells can become exhausted by the accumulation of active aldehydes, and suggests that scavenging or degrading compounds targeting the accumulation of aldehydes could prevent or reverse T cell exhaustion in the clinical setting ^[9]^. In doing so, caution will be required because aldehyde dehydrogenases that mediate aldehyde degradation are highly expressed in cancer stem-like cells, reduce oxidative stress, protect cancer cells from damage, and are associated with poor prognosis in human cancers ^[15]^. Managing the coexistence of cancer and T cells in the same microenvironment remains a major challenge in advancing cancer immunotherapy.

## Conflict of interest

The authors declare that they have no conflicts of interest.

## Funding

V.A.B. is supported by NIH grant (5R01CA257672-04).
